# Schwannoma diagnosed by endobronchial ultrasound‐guided intranodal forceps biopsy using standard‐sized biopsy forceps: A case report

**DOI:** 10.1111/1759-7714.15378

**Published:** 2024-05-25

**Authors:** Keigo Uchimura, Teruaki Ishida, Shumei Kan, Katsuhiko Aoyama, Akira Kisohara, Shingo Ikeda, Kohei Tagawa

**Affiliations:** ^1^ Department of Respiratory Medicine Kasukabe Medical Center Kasukabe Japan; ^2^ Department of Respiratory Medicine University of Occupational and Environmental Health, Japan Kitakyushu Japan; ^3^ Department of Thoracic Surgery Kasukabe Medical Center Kasukabe Japan; ^4^ Department of Thoracic Surgery Mitsui Memorial Hospital Chiyoda‐ku Japan

**Keywords:** endobronchial ultrasound‐guided intranodal forceps biopsy, endobronchial ultrasound‐guided transbronchial needle aspiration, mediastinal tumor, neurogenic tumor, schwannoma

## Abstract

Schwannomas are classified as neurogenic tumors and are the most frequent nerve sheath tumors in the paravertebral mediastinum. Recently, the addition of endobronchial ultrasound‐guided intranodal forceps biopsy (EBUS‐IFB) using standard‐sized biopsy forceps (SBFs) to endobronchial ultrasound‐guided transbronchial needle aspiration (EBUS‐TBNA) for metastatic lymph nodes in lung cancer patients reportedly improved the quality and quantity of the obtained specimens without significant complications. However, reports on the usefulness of this technique for benign diseases remain scarce. Here we report a case of schwannoma in the middle mediastinum, which was diagnosed by EBUS‐IFB using SBFs, despite inadequate specimens obtained via EBUS‐TBNA. An 80‐year‐old woman presented with dyspnea and a 5‐cm sized middle mediastinal tumor. EBUS‐TBNA and EBUS‐IFB using SBFs were performed for histological diagnosis. No complications were associated with the bronchoscopy procedure, and schwannoma was solely diagnosed using the EBUS‐IFB specimens. EBUS‐IFB using SBFs is potentially useful for diagnosing benign diseases, including schwannomas, which are often difficult to diagnose with EBUS‐TBNA.

## Graphical abstract

We report a case of schwannoma in the middle mediastinum, which was diagnosed by endobronchial ultrasound‐guided intranodal forceps biopsy (EBUS‐IFB) using standard‐sized biopsy forceps (SBFs). Schwannoma was solely diagnosed using the EBUS‐IFB specimens. EBUS‐IFB using SBFs is potentially useful for diagnosing benign diseases, including schwannomas, which are often difficult to diagnose with endobronchial ultrasound‐guided transbronchial needle aspiration.

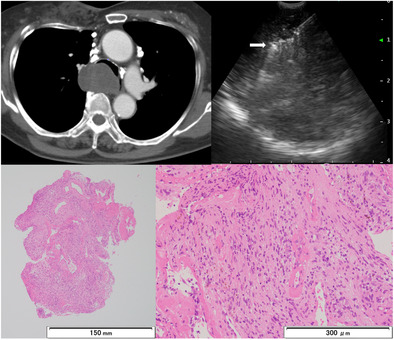

## INTRODUCTION

Schwannomas are classified as neurogenic tumors and are the most frequent nerve sheath tumors in the paravertebral mediastinum.^1^, ^2^ Patients with small tumors are usually asymptomatic, but subjective symptoms occur as the tumor grows and compresses adjacent vital organs.^2^, ^3^ Computed tomography (CT), magnetic resonance imaging, and positron emission tomography (PET)‐CT are used to diagnose schwannomas; however, biopsies such as endobronchial ultrasound‐guided transbronchial needle aspiration (EBUS‐TBNA), endoscopic ultrasound‐guided fine needle aspiration, and transthoracic biopsy (TTB) are necessary for a definitive diagnosis.^4^, ^5^ However, the small specimens obtained via EBUS‐TBNA and nonsurgical methods often lack sufficient tumor components, leading to false‐negative results.

Recently, the addition of endobronchial ultrasound‐guided intranodal forceps biopsy (EBUS‐IFB) with mini‐biopsy forceps (MBFs) or EBUS‐guided transbronchial mediastinal cryobiopsy (EBUS‐TMC) to EBUS‐TBNA for hilar and mediastinal lymphadenopathy reportedly improved the diagnostic yields.^6^, ^7^, ^8^ However, the high cost of disposable MBFs and cryoprobes is often a problem. Therefore, we devised a modified EBUS‐IFB technique using standard‐sized biopsy forceps (SBFs).^9^ The addition of EBUS‐IFB using SBFs to EBUS‐TBNA for metastatic lymph nodes in lung cancer patients reportedly improves the quality and quantity of specimens obtained, compared to that obtained using MBFs, without critical complications.^10^ However, reports on the usefulness of this technique for benign diseases remains limited.

Here, we report a case of schwannoma, which could only be diagnosed with EBUS‐IFB using SBFs.

## CASE REPORT

**FIGURE 1:**
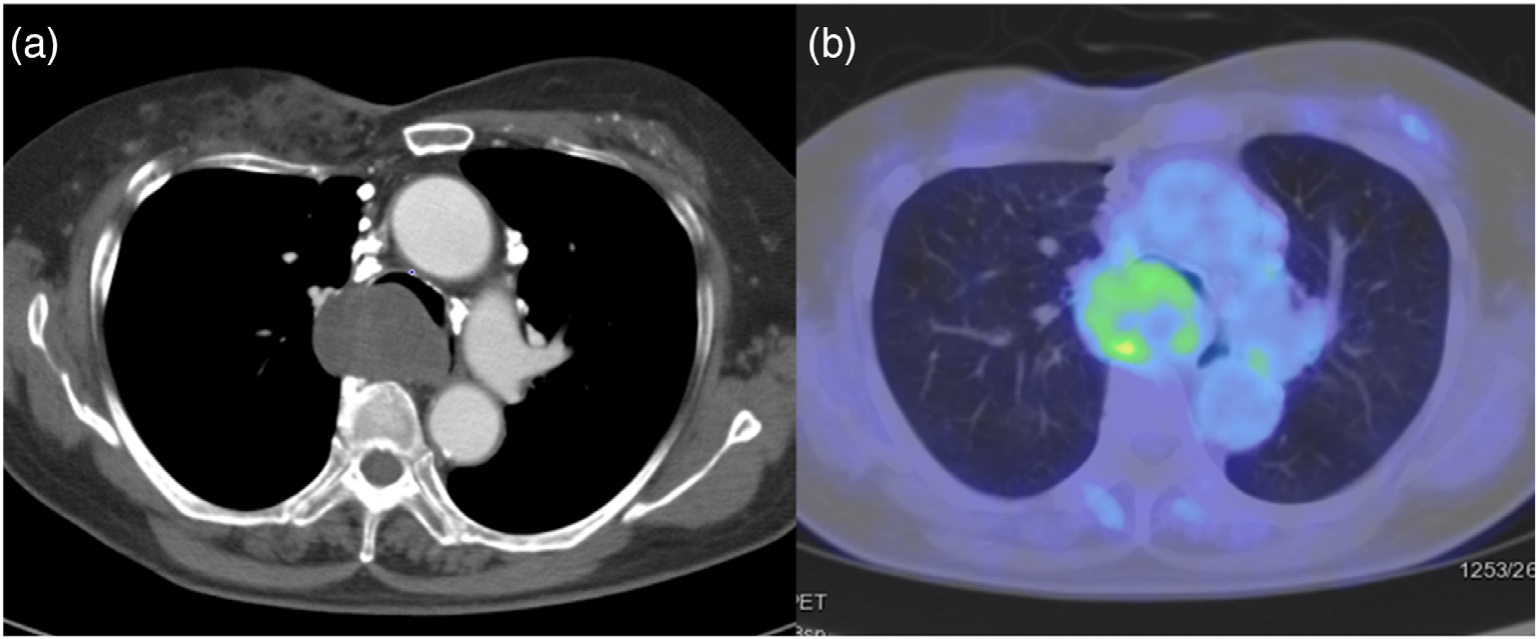
Chest computed tomography (CT) and positron emission tomography (PET)‐CT on the initial visit. (a) Chest CT showing a well‐circumscribed, homogeneous, 5‐cm sized middle mediastinal tumor (axial image). (b) PET‐CT showing a maximum standard uptake value of 3.4 on the mediastinal tumor (axial image).

**FIGURE 2:**
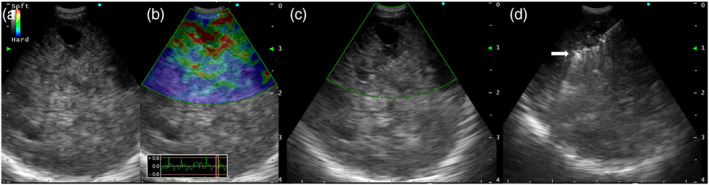
Bronchoscopic findings during diagnostic procedures. (a) Endobronchial ultrasound (EBUS) B‐mode image of the tumor displaying heterogeneous echogenicity, distinct margins, absence of central hilar structures, and presence of the coagulation necrosis sign. (b) EBUS elastographic image of the tumor showing partial blue. (c) EBUS image in power Doppler mode of the tumor showing reduced blood flow. (d) EBUS image during EBUS‐guided intranodal forceps biopsy (EBUS‐IFB) using standard‐sized biopsy forceps for middle mediastinal tumor (white arrow shows opened biopsy forceps within the tumor).

An 80‐year‐old woman with dyspnea on exertion was referred to our hospital for evaluation of a middle mediastinal tumor. She had no history of smoking but was on medication for hypertension and dyslipidemia. Her laboratory results were normal, including coagulation test results (Table 1). Chest CT revealed a 5‐cm sized middle mediastinal tumor (Figure 1a), which showed a maximum standard uptake value of 3.4 on PET‐CT (Figure 1b). For the diagnosis, EBUS‐TBNA was performed with four punctures, employing a convex probe ultrasound bronchoscope (BF‐UC290F, Olympus) and a 22‐gauge needle (Vizishot2, Olympus). This was followed by four EBUS‐IFB procedures using SBFs (FB‐231D, Olympus) after creating a tract with the “spiral digging technique” (Figure 2).^9^

**TABLE 1:** Patient's laboratory data on the initial visit.

Blood cell counts		Blood chemistry		Tumor marker	
WBC	7600/μL	TP	7.5 g/dL	CEA	1.7 ng/mL
Neutrophils	66.9%	T‐bil	0.5 mg/dL	CA19‐9	6.7 U/mL
Lymphocytes	26.1%	AST	26 IU/L	ProGRP	39.7 ng/mL
Eosinophils	0.3%	ALT	17 IU/L	SCC	1 ng/mL
Monocytes	6.1%	LDH	201 IU/L	HCGβ	1 ng/mL
Basophils	0.6%	ALP	61 IU/L	<Coagulation>	
RBC	4.50 × 10^6^/μL	γ‐GTP	16 IU/L	PT	12.2 s
Hb	13.1 g/dL	BUN	11.7 mg/dL	PT%	104%
Ht	39.8%	Cre	0.67 mg/dL	PT‐INR	0.93
Platelets	35.3 × 10^4^/μL	CRP	0.3 mg/dL	APTT	28.2 s

Abbreviations: ALP, alkaline phosphatase; ALT, alanine aminotransferase; APTT, activated partial thromboplastin time; AST, aspartate aminotransferase; BUN, blood urea nitrogen; CA19‐9, carbohydrate antigen 19–9; CEA, carcinoembryonic antigen; Cre, creatinine; CRP, c‐reactive protein; Hb, hemoglobin; HCGβ, human chorionic gonadotropin β subunit; Ht, hematocrit; INR, international normalized ratio; LDH, lactate dehydrogenase; ProGRP, pro gastrin releasing peptide; PT, prothrombin time; RBC, red blood cell; SCC, squamous cell carcinoma; T‐bil, total bilirubin; TP, total protein; WBC, white blood cell; γ‐GTP, gamma‐glutamyl transferase.

**FIGURE 3:**
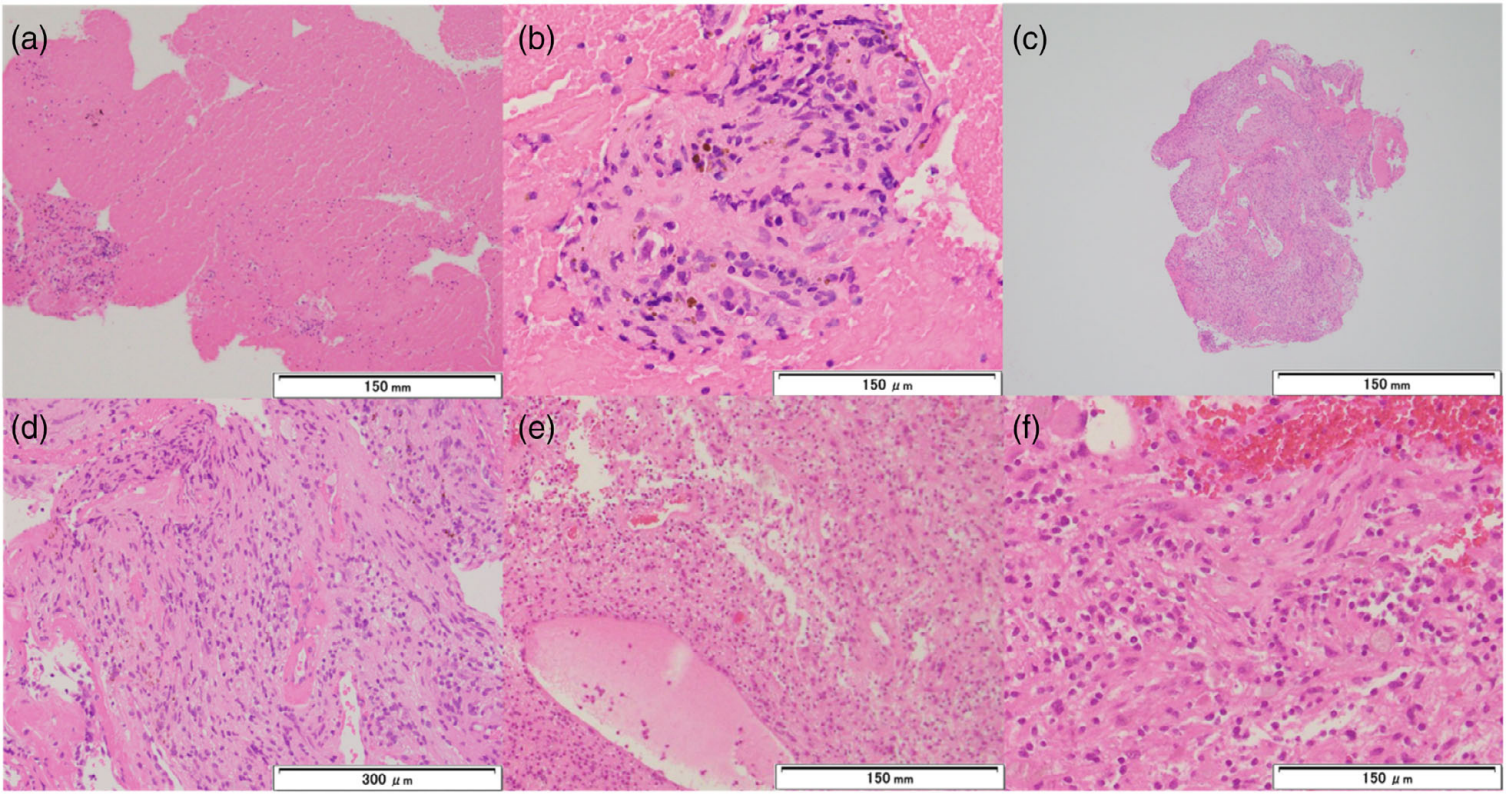
Histopathological findings of the specimens of the mediastinal tumor. (a, b) Endobronchial ultrasound‐guided transbronchial needle aspiration (EBUS‐TBNA) specimens mostly contained blood components with only a few spindle‐shaped cells. (c, d) Endobronchial ultrasound‐guided intranodal forceps biopsy (EBUS‐IFB) specimens revealed proliferation of spindle‐shaped cells arranged in an organized manner, with vitreous‐like thickened blood vessels and hemodyderosis in the background, indicative of schwannoma. (e, f) Surgical specimens revealed proliferation of spindle‐shaped cells arranged in an organized manner, similar to EBUS‐IFB specimens (a–f, hematoxylin and eosin staining).

The EBUS‐TBNA specimens contained mostly blood, with a few spindle‐shaped cells; however, this was insufficient for a definitive diagnosis (Figure 3a,b). Conversely, EBUS‐IFB specimens showed the proliferation of spindle‐shaped cells in an organized manner, with vitreous‐like thickened blood vessels and hemosiderosis in the background, indicative of schwannoma (Figure 3c,d). No complications were associated with the bronchoscopy procedure. The patient was transferred for surgery after diagnosis. The surgical specimens revealed the same pathological findings as the EBUS‐IFB specimens (Figure 3e,f).

## DISCUSSION

Here we present a case of schwannoma diagnosed with EBUS‐IFB using SBFs. Diagnosis of neurogenic tumors requires histopathological and imaging examinations, which can be challenging, with the exception of cases where surgical biopsy is conducted.^4^, ^5^ Although several case reports have previously reported the diagnosis of schwannoma using EBUS‐TBNA alone,^11^, ^12^, ^13^, ^14^, ^15^ a retrospective study of 86 neurogenic tumors of the mediastinum reported that the diagnosis was made in 84 cases using surgical specimens; two and three cases in which EBUS‐TBNA and TTB, respectively, were performed did not yield an accurate diagnosis of schwannoma.^3^ Herein, EBUS‐TBNA with four punctures yielded insufficient specimens for diagnosis, and the addition of EBUS‐IFB using SBFs was diagnostically efficacious.

Recently, the addition of EBUS‐IFB or EBUS‐TMC to EBUS‐TBNA for hilar and mediastinal lymphadenopathy reportedly improved the diagnostic yields, especially in uncommon tumors, lymphoma, and benign diseases;^6^, ^7^, ^8^ however, its usefulness has not been reported for schwannomas. The most commonly used forceps for EBUS‐IFB worldwide are 0.96 mm MBFs (M00515220, Boston Scientific),^6^ although there have been reports using 1.5 mm MBFs (FB‐433D, Olympus) and 1.9 mm SBFs (FB‐231D, Olympus).^9^, ^10^, ^16^

In a multicenter, prospective study of metastatic lymph nodes in lung cancer patients in Japan, EBUS‐IFB specimens obtained via 1.9 mm SBFs and 0.96 mm MBFs were compared.^10^ SBF specimens were three times greater than that obtained using 0.96 mm MBFs, and tissue architecture (tumor stroma) was better preserved with SBF specimens. Furthermore, SBF specimens showed less crush damage and blood contamination than MBF specimens, and forceps size notably affected specimen quality. Although studies regarding EBUS‐IFB using SBFs for benign diseases are lacking, these results suggest that EBUS‐IFB with SBFs may be useful for benign diseases, including schwannomas.

The advantage of EBUS‐IFB with SBFs is its lower cost compared to 0.95 mm MBFs or EBUS‐TMC. Bronchoscopic devices for biopsy are mostly disposable. In Japan, 0.96 mm MBFs and 1.1 mm cryoprobes are about five and 10 times more expensive than SBFs, respectively. Therefore, it is not feasible to routinely perform EBUS‐IFB with MFBs or EBUS‐TMC in addition to EBUS‐TBNA for hilar and mediastinal lymphadenopathy.

Of note, EBUS‐IFB with SBFs requires the creation of a larger tract to insert forceps compared to that of EBUS‐IFB with MFB. We experienced a case of metastatic lymph node dissemination through the tract into the trachea causing airway stenosis, 4 months after EBUS‐IFB with SBFs.^17^ Therefore, the safety of this procedure needs to be confirmed in a multicenter prospective study.

Recently, we have been able to use not only 22‐gauge needles but also larger 21‐ or 19‐gauge ones. Theoretically, a larger needle may allow for the collection of more abundant specimens, but no improvement in diagnostic yield has been reported with the use of 19‐ or 21‐gauge needles.^18^, ^19^, ^20^ Therefore, we believe that the EBUS‐IFB technique may be necessary for the diagnosis of some benign diseases.

In conclusion, we reported a case of schwannoma diagnosed by EBUS‐IFB using SBFs. EBUS‐IFB with SBFs may be useful for diagnosing benign tumors, including schwannomas.

## AUTHOR CONTRIBUTIONS

Keigo Uchimura drafted the manuscript. Keigo Uchimura, Teruaki Ishida, Shumei Kan, Katsuhiko Aoyama, Akira Kisohara, Shingo Ikeda and Kohei Tagawa were in charge of this patient. Teruaki Ishida, Shumei Kan, Katsuhiko Aoyama, Akira Kisohara, Shingo Ikeda and Kohei Tagawa helped to draft the manuscript. All authors read and approved the final manuscript.

## CONFLICT OF INTEREST STATEMENT

The authors declare that they have no competing interests. Written informed consent was obtained from the patient for the publication of this report.

## Data Availability

All data generated or analyzed during this study are included in this report. Further enquiries can be directed to the corresponding author.
